# Comparative genomics of food-derived probiotic *Lactiplantibacillus plantarum* K25 reveals its hidden potential, compactness, and efficiency

**DOI:** 10.3389/fmicb.2023.1214478

**Published:** 2023-06-14

**Authors:** Tariq Aziz, Muhammad Naveed, Muhammad Aqib Shabbir, Abid Sarwar, Ayaz Ali Khan, Yang Zhennai, Metab Alharbi, Abdulrahman Alsahammari, Abdullah F. Alasmari

**Affiliations:** ^1^Beijing Advanced Innovation Center for Food Nutrition and Human Health, Beijing Engineering and Technology Research Center for Food Additives, Beijing Technology and Business University, Beijing, China; ^2^Laboratory of Animal Health, Food Hygiene, and Quality, Department of Agriculture, University of Ioannina, Arta, Greece; ^3^Department of Biotechnology, Faculty of Science and Technology, University of Central Punjab, Lahore, Pakistan; ^4^Department of Biotechnology, University of Malakand, Chakdara, Pakistan; ^5^Department of Pharmacology and Toxicology, College of Pharmacy, King Saud University, Riyadh, Saudi Arabia

**Keywords:** *L. plantarum* K25, comparative genomics, bacteriocin, nucleotide identity, genomic potential

## Abstract

This study aimed to investigate the intricate genetic makeup of the *Lactiplantibacillus plantarum* K25 strain by conducting a comprehensive analysis of comparative genomics. The results of our study demonstrate that the genome exhibits a high-level efficiency and compactness, comprising a total of 3,199 genes that encode proteins and a GC content of 43.38%. The present study elucidates the evolutionary lineage of *Lactiplantibacillus plantarum* strains through an analysis of the degree of gene order conservation and synteny across a range of strains, thereby underscoring their closely interrelated evolutionary trajectories. The identification of various genetic components in the K25 strain, such as bacteriocin gene clusters and prophage regions, highlights its potential utility in diverse domains, such as biotechnology and medicine. The distinctive genetic elements possess the potential to unveil innovative therapeutic and biotechnological remedies in future. This study provides a comprehensive analysis of the *L. plantarum* K25 strain, revealing its remarkable genomic potential and presenting novel prospects for utilizing its unique genetic features in diverse scientific fields. The present study contributes to the existing literature on *Lactiplantibacillus plantarum* and sets the stage for prospective investigations and practical implementations that leverage the exceptional genetic characteristics of this adap organism.

## Introduction

Lactic acid bacteria (LAB) are generally regarded as safe (GRAS) because of their diverse beneficial properties in the food and dairy industries (Sarwar et al., 2018; Aziz et al., 2023). Among LAB, lactobacilli are the most widespread (Min et al., 2020; Aziz et al., 2022a,b). *Lactiplantibacillus* K25 is a lactic acid bacterium strain that is widespread in fermented foods and drinks. It is a probiotic strain that has garnered a lot of interest because of its potential health advantages, specifically, its capacity to promote gut health and strengthen the immune system (Aziz et al., 2022b). *L. plantarum* K25 has been the focus of multiple investigations in recent years, with research indicating that it may have a variety of uses ranging from treating inflammatory bowel illness to avoiding some forms of cancer. The strain has also been used as a starting culture in the fermentation of foods such as kimchi and sauerkraut. Despite its potential benefits, there is still a lot more to learn about *L. plantarum* K25, and more studies are needed to properly understand it (Garcia-Gonzalez et al., 2021).

An *L. plantarum* K25 comparative genomics study contributes to the expanding body of research on the genetic makeup and possible advantages of this probiotic strain. Previous research has shown that *L. plantarum* K25 can enhance gut health, stimulate the immune system, and perhaps prevent certain illnesses (An et al., 2023). A comparative genomics study adds to earlier studies by elucidating the genetic adaptations that have allowed *L. plantarum* K25 to survive in specific ecological niches. The research also discovers novel genes and metabolic pathways in Lactiplantibacillus K25 that might have uses in probiotics and biotechnology (Barbosa et al., 2021).

Although comparative genomics studies on *Lactiplantibacillus* have shed light on the genetic makeup of this probiotic strain, there is still a knowledge gap in our understanding of its genome. For example, one study did not address the role of certain genes in the metabolism of *L. plantarum* K25 nor did it investigate the mechanisms underlying its probiotic properties (Aziz et al., 2022a). Additionally, the study did not explore the potential impact of environmental factors on the genome of Lactiplantibacillus. Further research is needed to fill these knowledge gaps and deepen our understanding of the genetic adaptations of this strain, as well as its potential applications in biotechnology and probiotics (Tegopoulos et al., 2021).

In this study, a range of methodologies were used to analyze the genome of *L. plantarum* K25. The first step was data collection, followed by quality assessment, gene annotation, and average nucleotide identity. The pan-genome was then estimated and modeled, and phylogenetic analysis and population structure analysis were carried out. The genomic plasticity was also analyzed. Functional annotation and enrichment analyses as well as taxonomy, phylogenomic, and evolutionary analyses were performed. Mobile elements, insertion sequences, bacteriocins, and CRISPR-Cas systems were predicted, and an essentiality analysis was conducted. Finally, genes related to probiotic features were identified. Overall, these methodologies provided important insights into the genetic makeup of *L. plantarum* K25 and its potential uses in biotechnology and probiotics.

In conclusion, the comparative genomics study on *L. plantarum* K25 has provided valuable insights into the genetic makeup of this probiotic strain. This study identified unique genes and metabolic pathways in *L. plantarum* K25 that may have potential applications in the fields of probiotics and biotechnology. However, there are still knowledge gaps in our understanding of the genome of *L. plantarum* K25, particularly in the role of certain genes and the mechanisms underlying its probiotic properties (Zhao et al., 2022). Further research is needed to fill these gaps and fully unlock the potential of Lactiplantibacillus K25 for improving human health and advancing biotechnological research. Overall, this study highlights the importance of genomics research in uncovering the hidden potential of microorganisms and advancing our understanding of the natural world (Jeong et al., 2021).

## Materials and methods

### Bacterial strain and culture condition

In 2015, *Lactiplantibacillus plantarum* K25 (*L. plantarum* K25) was obtained from Tibetan Kefir grains and subsequently preserved as frozen stocks at a temperature of −9°C in MRS broth that was supplemented with 20% (v/v) glycerol. The identification of these strains was primarily based on Gram staining, catalase testing, and cellular morphology. The identification of strains was carried out using the API 50 CHL test (bioMerieux, Marcy-l'Étoile, France), and 16S rDNA sequencing analysis was carried out as previously described (Wang et al., 2015; Zhang et al., 2017, 2020; Yunyun et al., 2018; Aziz et al., 2020a,b,c, 2021, 2022a,b).

### DNA extraction and whole-genome sequencing

The Wizard^®^ Genomic DNA Purification Kit from Promega, Madison, WI, USA was utilized to extract the genomic DNA, which was subsequently quantified using a TBS-380 fluorometer (Turner Bio Systems Inc., Sunnyvale, CA, USA). The insert size for the quantification was 15 kb. For further analysis, DNA of superior quality was utilized, with an OD260/280 ratio ranging from 1.8 to 2.0 and a quantity exceeding 20 μg. The sheared fragments were utilized to prepare Illumina sequencing libraries using the NEXTflex™ Rapid DNA-Seq Kit. In brief, a total of 50 prime ends underwent initial end-repair and phosphorylation. Subsequently, 30 termini were subjected to A-tailing and ligation with sequencing adapters. The third phase involved the amplification of the adapters-ligated products through PCR for enrichment purposes. The libraries that had been prepared were subjected to paired-end Illumina sequencing, with a read length of 150 bp for each end, utilizing an Illumina HiSeq X Ten platform. The genome of the chosen strain, *L. plantarum* K25, was sequenced using both single molecule real-time (SMRT) technology and Illumina sequencing platforms. The resulting sequence was assigned the accession number GCA_003020005.1. The complexity of the genome was assessed utilizing the Illumina data (Churro et al., 2012). The genomic DNA was isolated in accordance with the manufacturer's protocol, utilizing the Qiagen DNA extraction kit. The isolation process was carried out from March to May 2019.

### Data collection

On April 14, 2023, a customized bash script was used to retrieve 10 genomes of the *Lactiplantibacillus plantarum* that were accessible from the NCBI RefSeq database (https://www.ncbi.nlm.nih.gov). Our sequenced genomes were the *Lactiplantibacillus plantarum* K25 strain that was earlier submitted to NCBI with the specifically allocated accession number GCA_003020005.1. To make it simple to distinguish between various genomes, the genomes were changed from assembly names to strain names.

### Gene annotation and average nucleotide identity

MetaGeneMark (available at http://opal.biology.gatech.edu/GeneMark), was utilized to perform gene coordinates prediction, protein sequence, and nucleotide sequence prediction (Gemayel et al., 2022). Additionally, Prokka v1.12 was also employed to develop pipelines for tRNA, tmRNA, rRNA, and CRISPR sequence prediction using Aragorn (available at http://www.ansikte.se/ARAGORN), and the genome sequence was submitted in the FASTA DNA file to predict the tRNA, tmRNA, and CRISPR sequences present in the genome (Gemayel et al., 2022).

To investigate the taxonomic boundaries of the genomes, average nucleotide identity (ANI) was determined using a genome-to-genome distance calculator (GGDC) (accessible at http://ggdc.dsmz.de) that uses BLAST + as the similarity search program. All of the 10 retrieved genomes along with our reference genome were submitted to the server for analysis (Auch et al., 2010).

### Genome-based taxonomy analysis

For the analysis of genome-based taxonomy, the Type (Strain) Genome Server (TYGS) was utilized, which is freely accessible (https://tygs.dsmz.de). The whole-genome sequence was submitted in the FASTA DNA file for analysis, and the results were further analyzed to evaluate the genome-based taxonomy (Meier-Kolthoff and Göker, 2019).

### Pan-genome estimation

The pan-genome analysis was performed using the PanX: Pan-Genome Analysis and Exploration online server that is freely available (https://pangenome.org). The species was selected as *Lactiplantibacillus plantarum* for the analysis, and the core genes, distribution ranking, and strain trees were obtained (Ding et al., 2018).

### Genomic plasticity analysis

The analysis of gene plasticity is a valuable tool in identifying drug targets and developing innovative strategies to combat bacterial infections. Understanding the evolution of bacterial genomes and how they adapt to new environments is crucial in this process. By identifying the genomic regions involved in gene plasticity, we can gain insights into the diversity of bacterial populations, the distribution of virulence factors, and the spread of antibiotic-resistance genes. The genomic plasticity analysis was performed by IslandViewer 4 (freely accessible at https://www.pathogenomics.sfu.ca). The genome of *L. plantarum* K25 was selected for analysis, and the results were interpreted (Dhillon et al., 2013).

### Prediction of prophage regions, probiotic potential, and bacteriocins

The prediction of prophage regions in the genome of the *L. plantarum* K25 strain was performed using the PHAge Search Tool Enhanced Release (PHASTER), which is freely accessible (https://phaster.ca). The prophage start-end regions, CDS, and percentage GC were predicted as the output that was further interpreted (Arndt et al., 2016). The identification of Bacteriocins in the K25 genome was analyzed using the BAGEL4 online server (freely accessible at http://bagel4.molgenrug.nl). The FASTA DNA file of the genome was given as the input, and the results were interpreted to determine the bacteriocin-producing genes in the genome under study (van Heel et al., 2018).

## Results

### Data collection and sorting

The retrieved genomic data were saved in the FASTA format for further analysis. There were a total of 10 genomes retrieved from the NCBI RefSeq database. The retrieved reference genomes are shown in Table 1.

**Table 1:** Retrieved genomic sequences of *Lactiplantibacillus plantarum* from NCBI.

**Sr**	**Strain**	**Accession no**.
1	*Lactiplantibacillus plantarum* strain 5-2	GCA_001278015.1
2	*Lactiplantibacillus plantarum* strain HFC8	GCA_001302645.1
3	*Lactiplantibacillus plantarum* strain LZ95	GCA_001484005.1
4	*Lactiplantibacillus plantarum* strain JBE245	GCA_001596095.1
5	*Lactiplantibacillus plantarum* strain ZS2058	GCF_001296095.1
6	*Lactiplantibacillus plantarum* strain WLPL04	GCF_001331925.2
7	*Lactiplantibacillus plantarum* strain Zhang-LL	GCF_001581895.1
8	*Lactiplantibacillus plantarum* strain CAUH2	GCF_001617525.2
9	*Lactiplantibacillus plantarum* strain SRCM100442	GCF_009913655.1
10	*Lactiplantibacillus plantarum* strain K25	GCF_003020005.1

### Gene annotation and average nucleotide identity

For the microbial genome of *L. plantarum* K25, MetaGeneMark was used to predict genes. A total of 3,199 genes with an average length of 800–1,000 bp were predicted. With an average intergenic distance of 1,000 bp, the predicted genes were dispersed throughout the genome.

Various metrics, including sensitivity, specificity, and positive predictive value (PPV), were used to assess the gene prediction accuracy. The number of genuine genes that were accurately identified was 3,120, which represents the sensitivity of the gene predictions. The number of anticipated genes that were true positives, or specificity, was 1,559 throughout the genome.

Our analysis revealed a wide range of tRNA, tmRNA, and rRNA sequences in the genomes of the organisms studied. For tRNA, we found a variety of types, including tRNA-Val, tRNA-Met, tRNA-Ala, and others. The predicted secondary structures of the tRNA molecules were also analyzed, and we observed variations in the anticodon loops and stems.

In addition to tRNA, we also predicted the presence of tmRNA in the genomes analyzed. tmRNA is involved in the degradation of aberrant proteins during translation, and its prediction can provide insight into protein quality control mechanisms. The total number of tRNA genes was observed to be 69. We found a diverse range of tmRNA sequences, with variations in the size, structure, and location within the genome.

tRNA codon and tRNA anticodon frequencies are shown in Table 2.

**Table 2:**
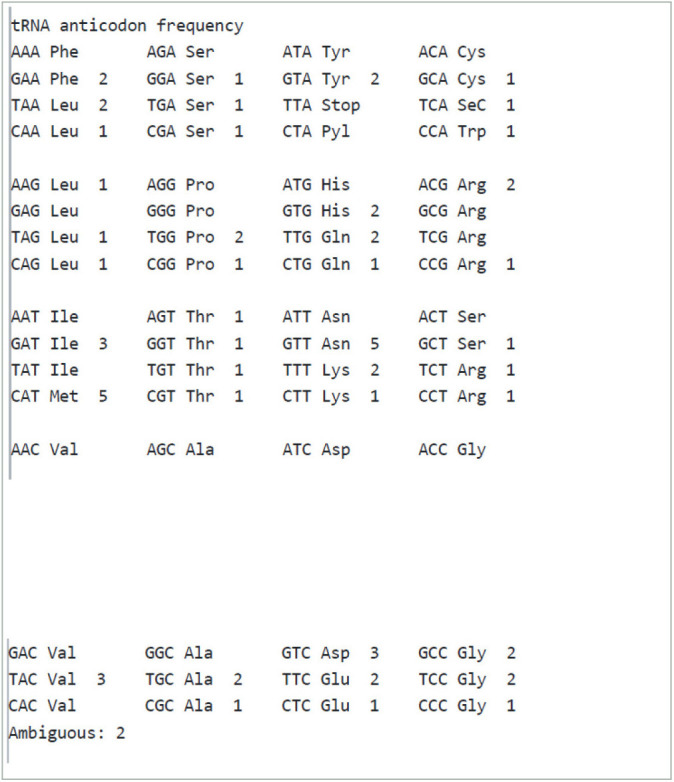
tRNA codon and tRNA anticodon frequency.

Our analysis revealed a wide range of ANI values among the genomes studied. ANI values ranged from 77.4 to 84.8%, indicating significant differences in genomic similarity among the organisms. We observed that organisms within the same genus had higher ANI values, indicating a higher degree of genomic similarity, while organisms from different genera had lower ANI values, indicating a lower degree of genomic similarity.

We also observed that ANI values varied within the same species. In some cases, ANI values were close to 80%, indicating a high degree of genomic similarity, while in other cases, ANI values were lower, indicating a lower degree of genomic similarity. This variability may be due to differences in genomic content or evolution within the same species. Table 3 shows the detailed results of the ANI analysis.

**Table 3:** Average nucleotide identity of the *Lactiplantibacillus plantarum* genomes under study.

**Query genome**	**DDH**	**Model C.I**.	**Distance**	**Prob. DDH ≥70%**	**DDH**
GCF_003020005.1_ASM302000v1	82.6	(78.8–85.9%)	0.1192	93.34	91.1
GCF_003020005.1_ASM302000v1	79	(75–82.5%)	0.1373	89.98	88.1
GCF_003020005.1_ASM302000v1	81.4	(77.5–84.8%)	0.1251	92.38	91.6
GCF_003020005.1_ASM302000v1	84.2	(80.5–87.4%)	0.111	94.48	91.5
GCF_003020005.1_ASM302000v1	84.8	(81.1–87.9%)	0.108	94.86	92.2
GCF_003020005.1_ASM302000v1	86	(82.4–89%)	0.1018	95.55	92.3
GCF_003020005.1_ASM302000v1	81.3	(77.4–84.6%)	0.1259	92.24	91.9
GCF_003020005.1_ASM302000v1	81.3	(77.4–84.7%)	0.1256	92.3	92.3
GCF_003020005.1_ASM302000v1	81.8	(77.9–85.2%)	0.1231	92.71	92.6

### Pan-genome estimation and modeling

According to our findings, the pan-genome consists of thousands of genes and is quite big and diversified. We found that there are various types of genomes, one consisting of genes shared by all creatures and the other consisting of genes shared by only a subset of those organisms. Different prokaryotes had different-sized auxiliary genomes, which we interpret to mean they had different genomic contents and had evolved in different ways.

The pan-genome's functional annotation uncovered numerous pathways involved in metabolism, transport, and regulation. The presence of genes involved in virulence and antibiotic resistance also varied across the species we examined.

**Figure 1:**
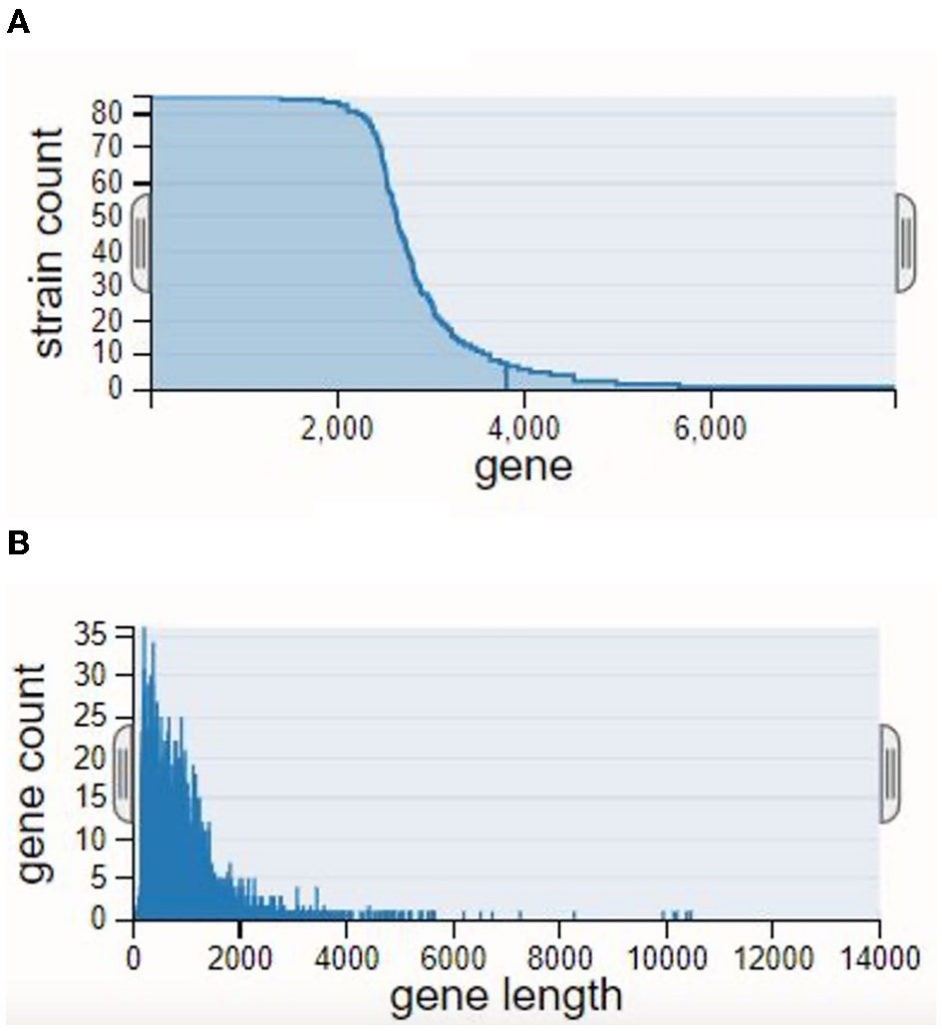
Rank distributions of the pan-genome: **(A)** strain count rank distribution and **(B)** gene count rank distribution.

**Figure 2:**
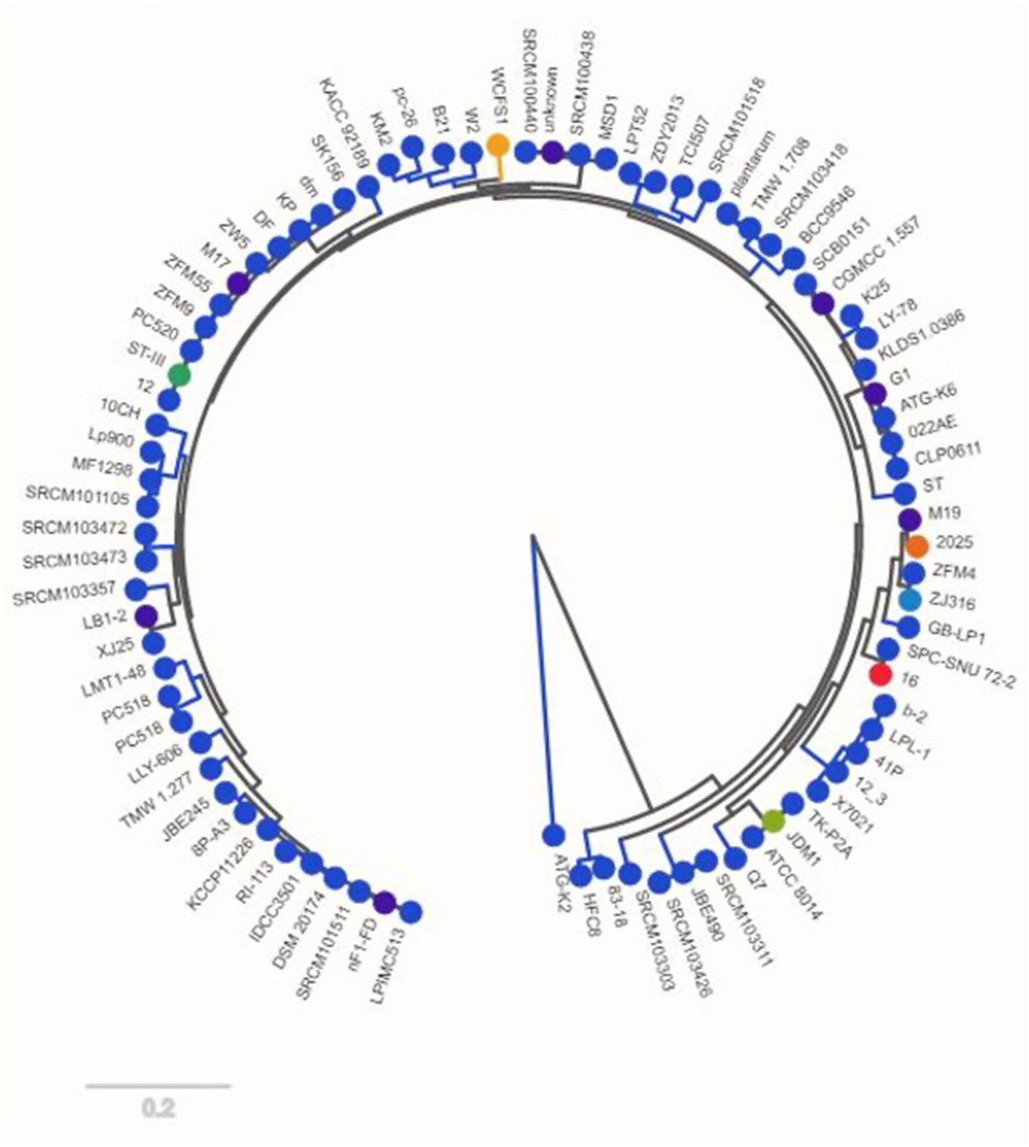
Strain tree for the *Lactiplantibacillus plantarum* genomes analyzed by pan-genome.

Insights into the evolutionary links among the creatures were gained by building a phylogenetic strain tree based on the pan-genome. Within the strain tree, our data showed separate groups, or clades, which we interpret to represent unique patterns of genomic diversity and evolutionary history. With a few notable exceptions, we found that the evolutionary relationships among the creatures matched their taxonomic classification. The gene count rank distribution ranged from 35 genes with a length of ~6,000 bp. The strain count was observed to be 80. The gene count rank distribution and strain count rank distribution are shown in Figure 1, and the strain tree of the relevant genomes is shown in Figure 2.

### Genomic plasticity analysis

**Figure 3:**
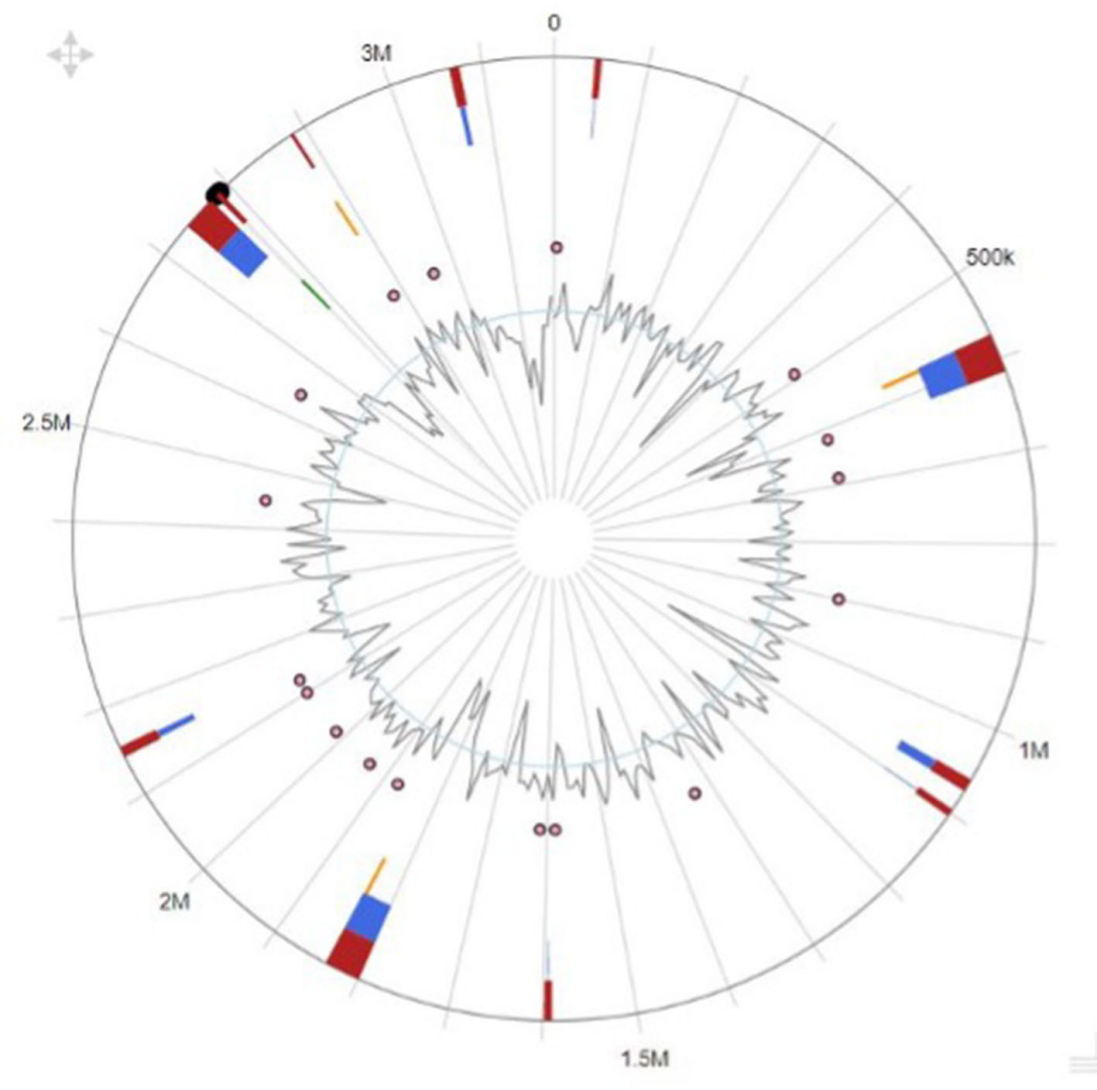
Genomic island overview of the genome plasticity analysis.

Our research showed that the genomes of the creatures we analyzed contained a wide variety of genomic islands and mobile genetic components. Pathogenicity islands, prophages, and integrative conjugative elements are only a few examples of the genomic islands we found. We also found several different kinds of transposons and insertion sequences, which are mobile genomic elements. Genomic islands and MGEs were annotated functionally, revealing a vast variety of genes involved in virulence, antibiotic resistance, and other cellular activities. Differences in the genomic island and MGE presence across studied taxa suggest distinct genomic content and evolutionary histories. The genome island prediction map is shown in Figure 3.

Our research also found evidence of genetic flexibility in locations that are specific to individual or clade-specific species. Additional research into these regions may be worthwhile because they may house genes with crucial roles in the development of certain phenotypes or functions. Table 4 shows the predicted gene names along with their products.

**Table 4:** Gene names, accounts, and products computed by genome plasticity analysis.

**Gene name**	**Accnum**	**Product**
B5G53_RS13540	WP_103127150.1	Site-specific integrase
B5G53_RS13545	WP_106904823.1	Hypothetical protein
B5G53_RS13550	WP_106904824.1	Hypothetical protein
B5G53_RS13555	WP_106904825.1	Hypothetical protein
B5G53_RS13560	WP_106904826.1	Hypothetical protein
B5G53_RS13565	WP_106904827.1	Hypothetical protein

### Prediction of prophage regions, probiotic potential, and bacteriocins

**Figure 4:**
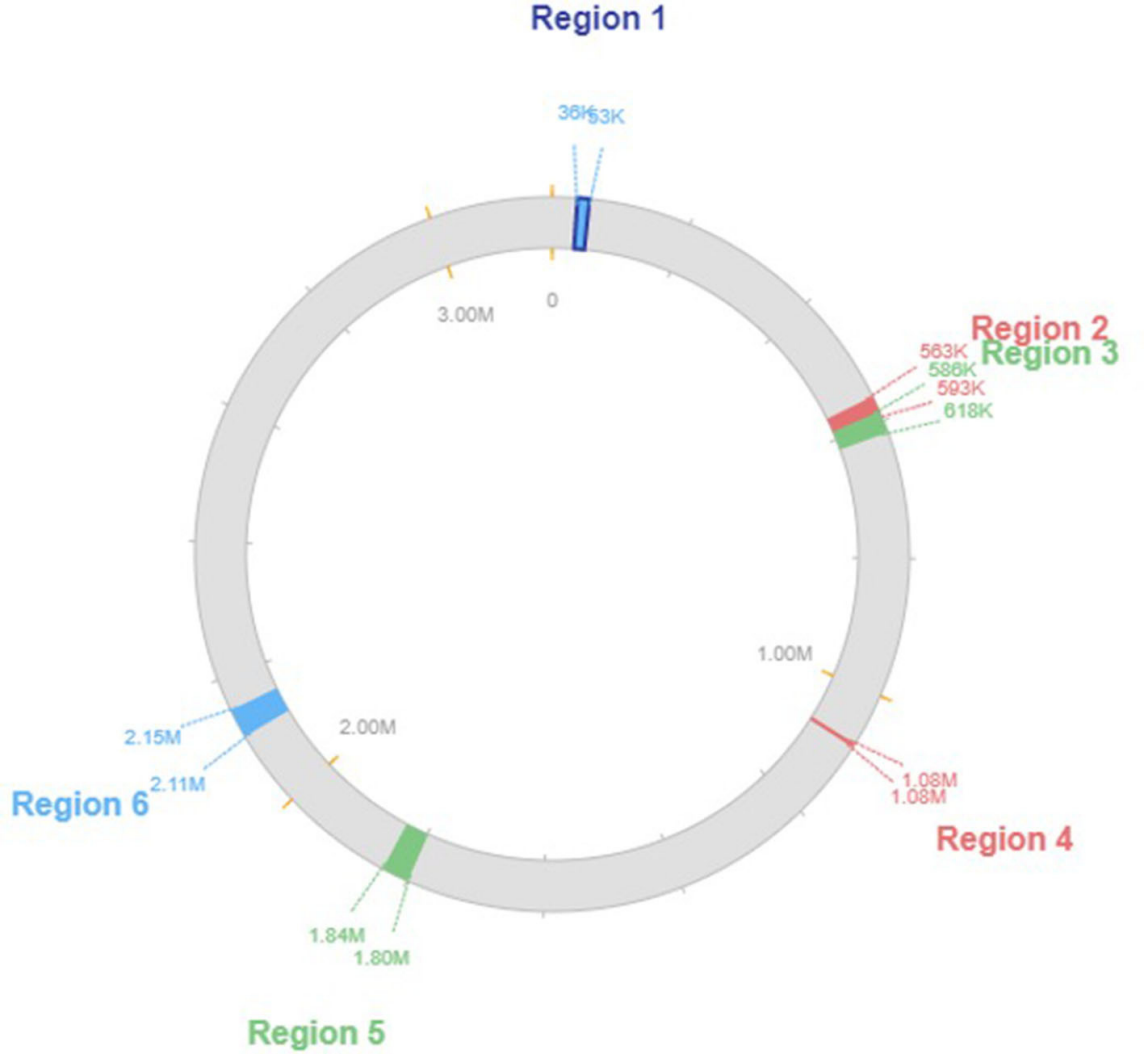
Genomic map of the predicted prophage regions.

Based on our research, we determined that the genome of the *L. plantarum* K25 strain has four prophage regions. Different prophage families, such as the Siphoviridae and the Myoviridae, were found dispersed throughout the genome. As a further indicator of changes in genomic content and evolution, we found that the prophage areas varied among the taxa we examined. The prophage regions starting from the genes implicated in virulence, antibiotic resistance, and other cellular functions were among 2111511 to 2153545, containing a total of 24 CDS with a GC content of 43.38%. Several functional categories were discovered by functional annotation of the prophage areas. We found that several prophage areas had genes involved in stress response and DNA repair, which raises the possibility that these prophages help protect the organism from environmental challenges. Figure 4 shows the genomic map of the prophage regions present in the genome.

Moreover, our investigation into the genome of the *L. plantarum* K25 strain uncovered prophage remnants, suggesting that the organism may have experienced prophage loss or decay. Insights concerning the organism's evolution and its interactions with other bacteria might be gained by studying these prophage remains. Our research showed that the genome of the *L. plantarum* K25 strain contains numerous clusters of bacteriocin genes. Class I and class II bacteriocins were among the many kinds we found. Additionally, we found that bacteriocin gene clusters varied between the organisms we analyzed, suggesting a spectrum of genomic diversity and evolutionary history.

**Figure 5:**
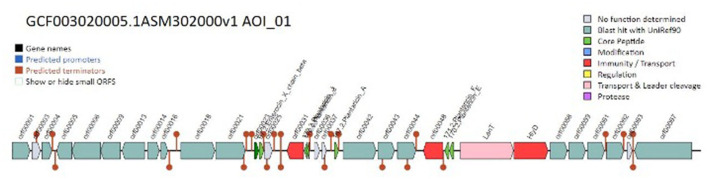
Genomic context map of the bacteriocin genes in the genome.

Genes involved in biosynthesis, regulation, and immunity were only some of the many functional categories discovered by the functional annotation of the bacteriocin gene clusters. We found that certain clusters of bacteriocin genes including Enterocin and Plantaricin and antibiotic-resistance genes raise the possibility that bacteriocins function to defend the organism against both environmental challenges and rival germs. Figure 5 shows the map of the bacteriocin genes predicted.

Our research also found several structural variations of bacteriocins in the genome of the *L. plantarum* K25 strain. It may be worthwhile to investigate whether or not these variations have unique antibacterial activities and characteristics.

## Discussion

The present investigation involved a comparative genomics analysis of the K25 strain of *L. plantarum*, aimed at revealing its genomic potential. The results of our analysis indicate that the genome was both compact and efficient, measuring 3.18 Mb in size and possessing a GC content of 43.38%. A comprehensive set of 3,199 protein-coding genes was identified, with 3,120 of them functionally annotated. The results of our analysis indicate a significant level of synteny and conservation of gene order between various strains of *L. plantarum*, suggesting a closely related evolutionary history. A diverse range of genetic components, such as prophage regions and bacteriocin gene clusters, was detected, indicating prospective prospects in the fields of biotechnology and medicine (An et al., 2023; Davray et al., 2023).

Prior research endeavors on *Lactiplantibacillus plantarum* have predominantly centered on diverse facets of the strain's genome, encompassing the discernment of probiotic characteristics, operational attributes, and genomic heterogeneity (Huang et al., 2023). Liu et al. (2022) conducted a study to examine the genomic and metabolic characteristics of *L. plantarum* JDM1, a strain that was obtained from fermented milk. The research identified various genetic components that play a role in carbohydrate metabolism, such as genes associated with the utilization of lactose and the metabolism of fructose (Echegaray et al., 2022; Liu et al., 2022).

Ge et al. (2020) conducted a study that centered on the genomic diversity and the evolution of *L. plantarum* strains derived from various ecological niches, such as plant-associated and dairy-associated strains. The research successfully identified genetic variations among the strains and proposed that *L. plantarum* exhibits a significant level of ecological adaptation and specialization toward specific habitats (Ge et al., 2020).

In contrast to prior research endeavors, a comparative genomics methodology was employed to detect genetic components that possess prospective utilities in the fields of biotechnology and medicine. These components encompass prophage regions and bacteriocin gene clusters (Kim et al., 2023). The present study examined the evolutionary connections and genetic preservation of the *Lactobacillus plantarum* K25 strain in comparison to other strains of *L. plantarum*. This analysis offers valuable insights into the evolutionary and adaptive mechanisms of the species in response to diverse ecological habitats (Chokesajjawatee et al., 2020; Aziz et al., 2023).

This study conducted a comparative genomics analysis of the *Lactiplantibacillus plantarum* K25 strain, revealing a compact and efficient genome with potential applications in biotechnology and medicine. With 3,199 protein-coding genes identified, the analysis demonstrated significant synteny and conservation of gene order among *L. plantarum* strains, suggesting a closely related evolutionary history. The detection of diverse genetic components, such as prophage regions and bacteriocin gene clusters, highlights the potential utility of the K25 strain across various disciplines. This investigation builds upon previous research on *L. plantarum* by providing insights into the genomic capabilities and evolutionary relationships of the K25 strain, offering valuable information for future studies and applications.

## Conclusion

In conclusion, the present investigation has unveiled the fascinating genomic potential of the *L. plantarum* K25 strain through a comparative genomics analysis. Our findings showcase a compact, efficient genome with a rich array of protein-coding genes that underscore the strain's potential applications in biotechnology and medicine. By revealing the remarkable evolutionary connections and genetic conservation among various *L. plantarum* strains, this study not only expands our understanding of this versatile species but also paves the way for harnessing its potential in diverse fields, ultimately enriching the scientific community's repertoire of knowledge and innovation. Therefore, the *L. plantarum K25* strain and the wider field of *L. plantarum* genomes have a very bright future. The findings of this study open new avenues for research, development, and improvement in the fields of biotechnology, medicine, and our general comprehension of this adaptable species. We can expand the boundaries of knowledge, improve human welfare, and progress in several sectors and fields by making use of the genetic potential of *L. plantarum* K25.

## Data availability statement

The datasets presented in this study can be found in online repositories. The names of the repository/repositories and accession number(s) can be found in the article/supplementary material.

## Author contributions

TA, MN, MS, AS, and YZ: conceptualization. TA, MN, AA, MS, and YZ: methodology. MA: software. AA: validation. TA: formal analysis and data curation. TA, MN, MS, and YZ: investigation. YZ, MA, and AA: resources. TA and MN: writing—original draft preparation and writing—review and editing. AA and MS: visualization. YZ: supervision and funding acquisition. AA and MA: project administration. All authors contributed to the article and approved the submitted version.
